# Thermal Properties of Cement-Based Composites for Geothermal Energy Applications

**DOI:** 10.3390/ma10050462

**Published:** 2017-04-27

**Authors:** Xiaohua Bao, Shazim Ali Memon, Haibin Yang, Zhijun Dong, Hongzhi Cui

**Affiliations:** 1 College of Civil Engineering, Shenzhen University, Shenzhen 518060, China; bxh@szu.edu.cn (X.B.); 2150150417@email.szu.edu.cn (H.Y.); h.z.cui@szu.edu.cn (H.C.); 2 Department of Civil Engineering, School of Engineering, Nazarbayev University, Astana 010000, Kazakhstan; shazim.memon@nu.edu.kz

**Keywords:** composite phase change materials, geothermal energy storage, cement-based composites, structural–functional integrated materials, mechanical properties, thermal properties

## Abstract

Geothermal energy piles are a quite recent renewable energy technique where geothermal energy in the foundation of a building is used to transport and store geothermal energy. In this paper, a structural–functional integrated cement-based composite, which can be used for energy piles, was developed using expanded graphite and graphite nanoplatelet-based composite phase change materials (CPCMs). Its mechanical properties, thermal-regulatory performance, and heat of hydration were evaluated. Test results showed that the compressive strength of GNP-Paraffin cement-based composites at 28 days was more than 25 MPa. The flexural strength and density of thermal energy storage cement paste composite decreased with increases in the percentage of CPCM in the cement paste. The infrared thermal image analysis results showed superior thermal control capability of cement based materials with CPCMs. Hence, the carbon-based CPCMs are promising thermal energy storage materials and can be used to improve the durability of energy piles.

## 1. Introduction

In recent years, the building division of China was one of the principal energy users and accounted for nearly 40% of total fossil energy [1]. The decline in fossil energy consumption and increase in proportion of renewable energy are pushing the designers as well as energy policy at regional, national and international levels, to adopt more energy-efficient technologies [2,3,4]. Consequently, building energy efficiency has become one of the main research areas [5,6,7].

**Figure 1:**
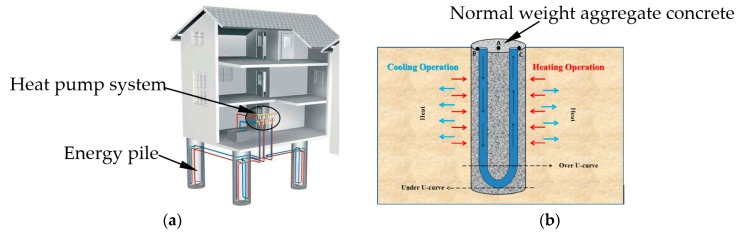
Energy pile application in building energy efficiency. (**a**) Schematic drawing of geothermal piles system [14]; (**b**) Heating/cooling operation of energy piles during summer/winter modes [15].

Geothermal energy piles are utilized to transport and store geothermal energy from the surrounding ground. Figure 1 shows schematic drawing of energy pile application in building energy efficiency. The large thermal mass of concrete piles can further be improved by the inclusion of phase change material (PCM). PCMs have been used in buildings for a long time and are considered as a potential candidate to solve issues related to thermal energy shortage by reducing the mismatch between thermal energy supply and demand in time and space. When PCM are combined with building materials, they increase the thermal energy storage capacity of building elements [8]. In this regard, direct and immersion techniques have been used to combine PCM with porous building materials [9]. However, the retention capacity of these methods is low and hence vacuum impregnation method is used to increase the encapsulation efficiency of porous materials [10]. Some researchers have presented excellent reviews on PCMs [9,11,12,13]. 

Many studies have been carried out to investigate geotechnical performance of piled foundations for ground-source heat-pump systems [16,17]. Application of PCMs into energy piles can help in maintaining a stable temperature and subsequently can reduce the axial load acting on the piles. Among PCMs, paraffin is usually preferred, as it is generally believed to be chemically inert, non-corrosive, innocuous, inexpensive, and recyclable and show small volume changes during phase transition. It is pertinent to mention here that the lower thermal conductivity of paraffin limits its application in energy storage [18]. However, in numerous investigations [11,12,13,14,15,16,17], PCMs thermal conductivity was enhanced by using expanded graphite (EG) and GNPs (Graphene nanoplatelet, GNP) because they have superior thermal conductivity, low density and are environmental friendly and safe. 

Various researchers have investigated the mechanical properties as well as heat of the hydration of the thermal energy storage cement-based composites. Zhang et al. [19] prepared cement mortar for thermal energy storage (TES) by incorporating n-octadecane/expanded graphite with percentage varying from 0.5 to 2.5 wt %. When compared to control TES cement mortar, the compressive strength of TES cement mortar with 2.5 wt % n-octadecane/expanded graphite was reduced by 55%. However, because of the excellent thermal performance, the authors concluded that composite can be used for TES applications. Hunger et al. [20] prepared TES self-compacting concrete by incorporating microencapsulated PCM (1, 3, 5 wt %). Test results showed that, by incorporating 5% microencapsulated PCM, the temperature peak of hydration was reduced by up to 28.1%. The reduction in compressive strength with 5% microencapsulated PCM was up to 69%. However, the authors reported that the compressive strength with 3% microencapsulated PCM was acceptable for most constructional purposes. The mechanical properties of TES cement-based composite were evaluated by incorporating paraffin/diatomite (10%, 15%, 20%, and 30% by weight of cement) [21]. In comparison to control cement-based composite, the compressive and flexural strength (at 28 days) of TES cement-based composite with 30 wt % paraffin/diatomite was reduced by 48.7% and 47.5%, respectively. The prepared samples showed good thermal energy storage performance as depicted by specific heat capacity results. Bentz and Turpin [12] evaluated the performance of PCM incorporated cement mortars by testing it in semi-adiabatic conditions. From test results, it was found that PCM lowered the peak temperature by about 8 °C while the delay in the peak temperature was little bit more than an hour. The authors concluded that PCM strongly influenced the heat evolution in cement mortars.

In this research, two kinds of composite phase change materials i.e., expanded graphite-based PCM (EG-Paraffin) and graphite nanoplatelet-based PCM (GNP-Paraffin) were prepared for the development of cement-based structural–functional integrated materials. The thermo-regulating performance of thermal energy storage cement-based composite by using infrared thermography was also determined. Finally, the role of CPCM, namely, expanded graphite-based PCM (EG-PCM) and graphene nanoplatelet-based PCM (GNP-PCM), in reducing the heat of the hydration of the cement-based composite was evaluated.

## 2. Materials and Methods 

### 2.1. Materials

Industrial grade paraffin from China Petrochemical Corporation was used as PCM, while expanded graphite (particle size 0.5 mm, expansion ratio 300) purchased from Qingdao Teng Sheng Da Carbon Machinery Co., Ltd., Qingdao, China, and technical-grade graphene nanoplatelets (a diameter of 5–7 μm and a nanosheet number <20) purchased from Chinese Academy of Sciences Chengdu Organic Chemical Co., Ltd., Chengdu, China, were used as supporting materials. For the preparation of CPCM cement paste, Portland cement (P.II 52.5R from Guangdong huarun cement Co., Ltd., Guangzhou, China) conforming to BS 12-1996 (Specification for Portland cement) was used (a 28-day compressive strength of 60.1 MPa). The specific gravity of cement was 3.44 g/cm^3^, while the specific surface area of cement was 3980 cm^2^/g. The details of the chemical composition of the ordinary Portland cement (OPC) are specified in Table 1.

### 2.2. Preparation of CPCMs

Two kinds of CPCMs, i.e., EG-Paraffin and GNP-Paraffin, were prepared using vacuum impregnation [22,23]. Considering the PCM leakage as mentioned in [24], in this research, the vacuumed CPCM was kept on the filter paper to remove surplus paraffin. Thereafter, the CPCM was placed in an oven at 80 °C for at least three days, and the percentage of PCM retained by EG or GNP was determined. During the heating period, the high absorption paper was changed until the CPCM mass became constant. This method can eliminate CPCM expansion as well as the PCM leakage. 

### 2.3. Preparation and Mix Proportion of CPCMs Cement Paste Specimens

It is known that PCM require a larger specific surface area to exchange heat efficiently. Hence, CPCM was added into a cement paste matrix rather than partially replacing aggregates in mortar or concrete. The details of mix proportions are shown in Table 2. The preparation procedure is as follows. Firstly, cement and CPCM were dry mixed for 1 min at a low speed. Thereafter, the mixture of superplasticizer and water was added, and mixing continued at a low speed for 3 min followed by high-speed mixing of constituents for 1 min. The obtained CPCM cement paste was compacted on a vibration table. Thereafter, in order to prevent water loss, the samples were covered with a plastic sheet. The samples were then demolded after 24 h. Finally, the samples were cured for 28 days at 20 ± 1 °C temperature and 99% relative humidity. For this research, 40 × 40 × 160 mm^3^ prisms (for the flexural test), 20 × 20 × 20 mm^3^ cubes (for compressive strength), and Ф 30 mm × 4 mm cylinders (for infrared thermography) were prepared.

### 2.4. Test Methods Used for the Characterization of CPCMs

#### 2.4.1. Thermal Properties of CPCMs

The thermal properties of the CPCMs (DSC-Q200, TA Instruments, Newcastle, PA, USA) were determined by using DSC (under nitrogen atmosphere). The parameters used for testing are as follows: temperature range: 0–60 °C; heating/cooling rate: 2 °C /min; flow rate: 40 mL/min.

#### 2.4.2. Environmental Scanning Electron Microscopy (ESEM)

The SEM images of cement pastes were captured using ESEM (Quanta 250 FEG, FEI Company, Hillsboro, OR, USA). The machine was operated in secondary-electron detection mode (under low vacuum) at an accelerating voltage of 15 kV. In order to get representative images, several regions of the powdered samples were observed. The SEM micrographs were also captured for thermal energy storage cement paste, while the energy dispersive spectrometer (EDS) was used to evaluate CPCMs dispersion in cement paste. Since PCM is absorbed by carbon-based CPCM and there is no carbon element present in pure cement paste, the EDS map showing distribution of carbon element can be used to represent the distribution of EG-Paraffin/GNP-Paraffin in CPCM.

#### 2.4.3. Mechanical Properties of Thermal Energy Storage Cement Composites

At 28 days, the mechanical properties (compressive and flexural strength) of the cement composite with 0%, 10%, and 20% CPCMs (EG-PCM and GNP-PCM) by weight of cement were determined. The loading rate for compressive strength was 2400 ± 200 N/s, while it was 50 ± 10 N/s for flexural strength.

#### 2.4.4. Infrared Thermography of Thermal Energy Storage Cement Composites

Infrared thermography has been used sucessfully to determine the thermal-regulating performance of CPCMs cement paste [25]. Hence, in this research, the thermal-regulating performance of CPCMs cement paste was evaluted by using infrared thermography with an infrared thermal imager (FLIR T440; USA). For this purpose, the disk specimens (Ф 30 mm × 4 mm) with 0%, 10%, and 20% CPCMs (EG-paraffin and GNPs-Paraffin) by weight of cement were perpared. A PTFE disk (Ф 30 mm) with a 5 mm thickness was placed in between the test sample and hot plate so as to avoid the sample’s temperature from rising too quickly. For mointoring, the microscope lens was placed at 30 cm from the upper surface of the sample. Before testing, the sample was cooled to 3 °C and then kept on the hot plate at a constant temperature (40 °C). Finally, the video was exported from the infrared thermography and image processing was done using photoshop. 

#### 2.4.5. Hydration Heat of Thermal Energy Storage Cement Composites

The heat of the hydration of the cement paste with and without CPCM was evaluated. The control cement paste and the cement paste containing 25% CPCM (EG-paraffin and GNPs-Paraffin) was made with a water/cement ratio of 0.35. The heat of the hydration of the CPCM cement paste was evaluated for the first 72 h using ToniCal Trio 7338 (Toni Technik, Zwick/Roell Group, Berlin, Germany).

## 3. Results and Discussion

### 3.1. SEM of CPCMs

**Figure 2:**
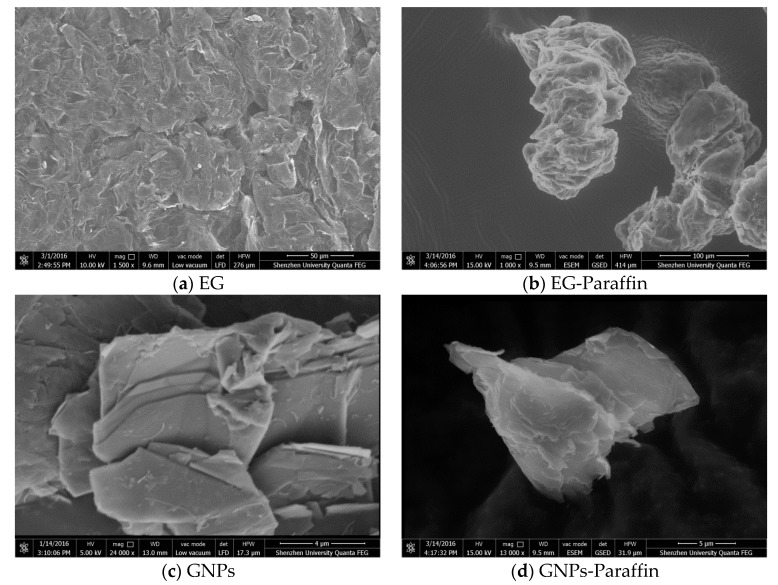
SEM micrographs of (**a**) expanded graphite (EG); (**b**) EG-Paraffin CPCM; (**c**) graphene nanoplatelets (GNPs); and (**d**) GNPs-Paraffin CPCM.

The SEM micrographs of EG, GNPs, EG-Paraffin CPCM, and GNPs-Paraffin CPCM are shown in Figure 2. Paraffin was held by a honeycomb network of EG due to capillary and surface tension forces while it was held by flaky GNP particles (Figure 2c,d) due to their tendency to absorb organic materials on their surface [26].

### 3.2. Thermal Properties of CPCMs

**Figure 3:**
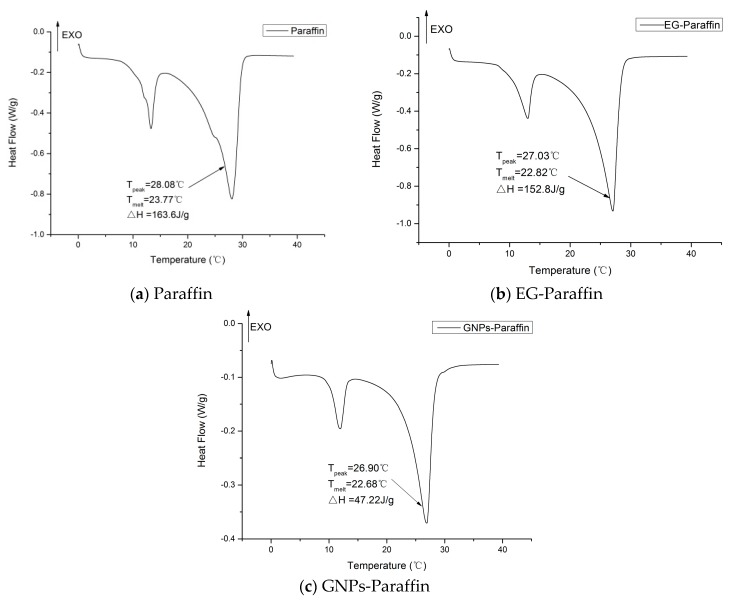
DSC curves—Paraffin and CPCMs. (**a**) Paraffin (**b**) EG-Paraffin and (**c**) GNPs-Paraffin.

The thermal properties of paraffin and CPCMs are presented in Figure 3. The melting temperatures for paraffin, EG-Paraffin, and GNP-Paraffin were 23.77 °C, 22.82 °C, and 22.68 °C, respectively. This shows that, with the incorporation of EG and GNP, the melting point of the PCM decreased. The decrease in the melting temperature is believed to be due to higher thermal conductivities of EG and GNP, which in turn resulted in the increase in heat transfer. The latent heats of melting for paraffin, EG-Paraffin, and GNP-Paraffin were 163.6 J/g, 152.8 J/g, and 47.22 J/g, respectively. By using Equation (1), the encapsulation efficiency for EG-Paraffin and GNP-Paraffin was determined as 93.51% and 30.02%, respectively. The latent heat values obtained in this research are higher than those reported in the literature [27,28]. Therefore, the developed CPCMs are promising thermal energy storage candidates and can be used for energy piles and buildings.

η(%) = (ΔH_m,EG-PCM/GNP-PCM_)/(ΔH_m,PCM_) × 100% (1)

It is pertinent to mention here that the developed CPCMs were found to be chemically compatible, thermally stable, and reliable.

### 3.3. Mechanical Properties of Thermal Energy Storage Cement-Based Composites

The compressive strength results of cement composites are presented in Table 3. With the increase in the percentage of CPCM in the mix, the compressive strength of TES cement paste decreased. For 10% and 20% replacement level, the percentage decrease for EG-Paraffin thermal energy storage paste was found to be 77.9% and 86.4%, respectively. In comparison to EG-Paraffin, the percentage decrease in compressive strength for GNP-Paraffin thermal energy storage cement paste was lower and was found to be 44% and 61.3% for 10 wt % and 20 wt % cement replaced by GNP-Paraffin composites. In Zhang et al.’s research [19], the percentage reduction in the compressive strength of cement mortar cubes containing 2.5% n-octadecane/EG CPCM was found to be 55%. Xu et al. [21] determined the compressive strength of TES cement-based composites incorporated with paraffin/diatomite (by weight of cement). At 28 days, the compressive strength of TES cement-based composite with 30 wt % paraffin/diatomite was reduced by 48.7% when compared with control cement-based composites [21]. Xu et al. [29] also determined the compressive strength of TES cement-based composites by incorporating paraffin/expanded vermiculite (0%, 50%, and 100% by volume) as a fine aggregate replacement. The compressive strength of TES cement-based composite with 50% and 100% paraffin/expanded vermiculite was reduced by 47.4% and 56.5%, respectively. For TES self-compacting concrete incorporated with 5 wt % microencapsulated PCM, the compressive strength was reduced by up to 69% [20]. However, the authors reported that the compressive strength with 3% microencapsulated PCM was acceptable for most constructional purposes. Fernandes et al. [30] used paraffin-based microencapsulated PCM for the development of cement-based composites. The authors found that compressive strength was reduced when an increased quantity of microencapsulated PCM (0%, 10%, and 20% by volume of paste fraction) was used. The decrease in compressive strength values with 20% microencapsulated PCM was found to be approximately 40% at a water/cement ratio of 0.35 and approximately 50% at a water/cement ratio of 0.45. Hence, the reduction in compressive strength was dependent on the water/cement ratio. Nonetheless, in this research, the compressive strength of the thermal energy storage cement paste with 20 wt % cement replaced by GNP-Paraffin composites (C-GNP/PCM-20) was 25.6 MPa and is acceptable for many applications, as reported in the literature [19,21,31,32] and the Chinese National standard (GB 50574-2010) for building materials. 

The flexural strength of thermal energy storage cement paste was also determined. The flexural strength decreased with the addtion of CPCM in the cement paste. For 10 wt % and 20 wt % replacement level, the percentage decrease for EG-Paraffin thermal energy storage paste was found to be 73.4% and 83.5%, respectively, while the percentage decrease for GNP-Paraffin thermal energy storage cement paste was found to be 31.64% and 41.8%, respectively. In Xu et al.’s research [21], the flexural strength (at 28 days) of TES cement-based composite with 30 wt % paraffin/diatomite was reduced by 47.5% when compared to the control TES cement-based composite. In another study by Xu et al. [29], the flexural strength of TES cement-based composite with 50% and 100% paraffin/expanded vermiculite used as a replacement (by volume) of fine aggregate was reduced by 23.3% and 21.9%, respectively. 

The density values of the thermal energy storage cement paste is shown in Table 4. The density decreased with the increase in the percentage of CPCM. For 10% and 20% replacement level, the percentage decrease in density for EG-Paraffin thermal energy storage paste was found to be 27.81% and 29.76%, respectively, while the percentage decrease for GNP-Paraffin thermal energy storage paste was found to be 7.02% and 14.33%, respectively. For a similar replacement level and in comparsion to GNP-Paraffin thermal energy storage cement paste, the percentage decrease in density for EG-Paraffin thermal energy storage cement paste was higher. It is believed to be due to the porous nature of expanded graphite. Xu et al. [29] found that the density of lightweight TES cement-based composites incorporated with 50% and 100% paraffin/expanded vermiculite as a replacement (by volume) of fine aggregate was reduced by 20.1% and 24.7%, respectively. Conclusively, the thermal energy storage cement-based composites can meet structural requirements for energy pile application.

**Figure 4:**
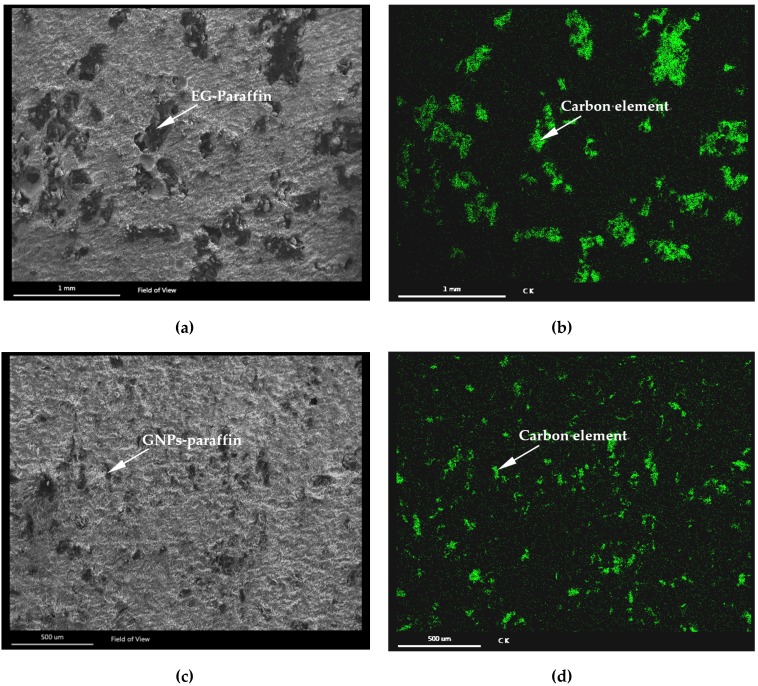
SEM image of cement paste containing two different phase change materials (PCMs): (**a**) cement paste containing 10% of EG-Paraffin; (**b**) EDS for EG10; (**c**) cement paste containing 10% of GNPs-paraffin; (**d**) EDS for GNPs10. (Carbon elements are shown in green).

Figure 4 shows the morphology of EG-Paraffin and GNP-Paraffin thermal energy storage cement pastes along with element mapping, and shows the dispersion of EG-Paraffin and GNP-Paraffin in the cement paste. It can be seen that EG-Paraffin and GNP-Paraffin are well dispered in the cement paste, depicting uniform mixing of CPCM during the mixing stage. This would help the geothermal energy in the piles to be evenly stored.

### 3.4. Infrared Thermal Imaging Analysis

**Figure 5:**
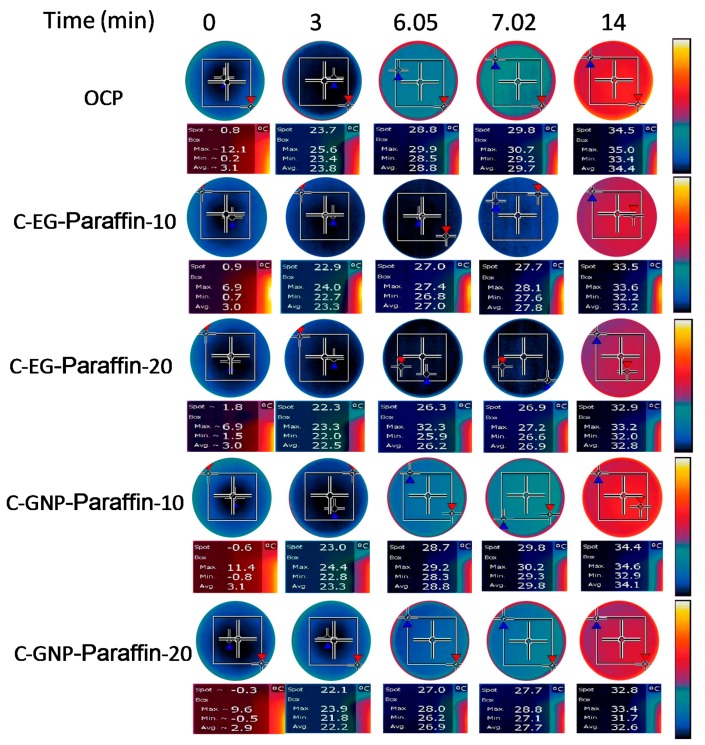
Infrared thermograph images of the cement paste disks with and without CPCMs heated for different time periods. The maximum, minimum, and average temperatures and the temperatures at the marked point (Spot) are presented under each thermal image.

The results of the surface temperature distribution of cement paste with and without CPCMs at various times are shown in Figure 5, while the temperature difference between cement pastes with and without CPCMs is tabulated in Table 4. In general, the surface temperatures of specimens incorporated with CPCMs are lower than ordinary cement paste (OCP) at different stages. This phenomenon exactly illustrates the thermal control capacities of cement pastes combined with CPCM. At 6.05 min, the two specimens (C-EG-Paraffin-10 and C-GNP-Paraffin-20) simultaneously reached peak temperatures. From the DSC test, it is known that the peak temperature of EG-Paraffin and GNP-Paraffin is 27.03 °C and 26.9 °C, respectively. It means that C-EG-Paraffin-10 and C-GNP-Paraffin-20 absorb the same amount of thermal energy from 0 to 6.05 min. In fact, the quantity of paraffin in C-EG-Paraffin-10 (9.3 wt %) and C-GNP-Paraffin-20 (6.2 wt %) is different. This shows that the lower quantity of PCM in C-GNP-Paraffin-20 can have a similar capacity of controlling temperature when compared to C-EG-Paraffin-10. The reason for this is believed to be a different thermal conductivity coefficient of EG and GNP. According to [33], the thermal conductivity coefficient of graphene is as high as 3000–5000 W/(m·K), while, in contrast, the thermal conductivity coefficient of expanded graphite is about 500 W/(m·K), i.e., the thermal conductivity coefficient of GNP is about 6–10 times higher than that of EG. This means that the higher thermal conductivity coefficient of GNP makes the PCM in GNP-Paraffin absorb the heat efficiently. It can also be seen from Table 4 that the heat absorption capacity almost reaches the maximum value at 6.05 min, i.e., the surface temperature of specimens did not change much from 6.05 to 7.02 min. This is due to the reason that PCMs have already melted. At 7.02 min, the average temperature difference for C-EG-Paraffin-20 and C-EG-Paraffin-10 was reduced by 2.8 °C and 2.0 °C, respectively. At this stage, the advantage of a high absorption capacity of EG was prominent, i.e., C-EG-Paraffin-20 delayed the peak temperature by about 1 min when compared with C-GNP-Paraffin-20. At 14 min, it can be seen that temperature reduction capacity has no significance, as reduction in temperature difference between cement paste with and without CPCMs was about 1 °C. This shows that the temperature controlling capacity of the CPCM gradually reduces when the temperature exceeds the melting point of PCM.

### 3.5. Hydration Heat of TESCP

**Figure 6:**
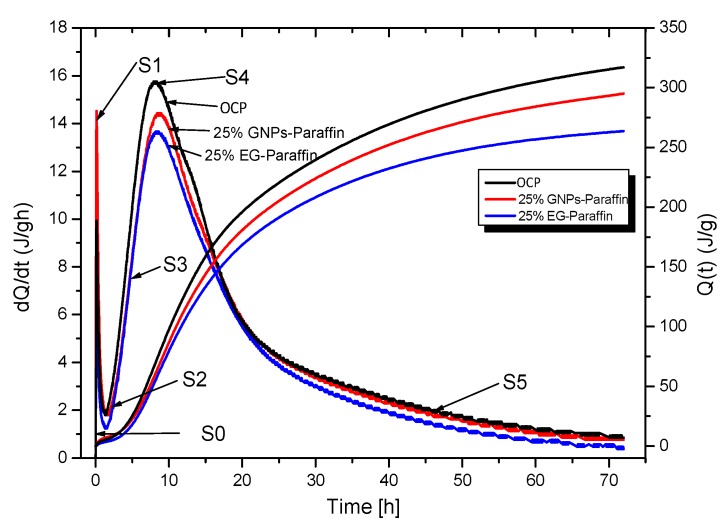
The effect of PCM addition on amount *Q*(t) of the heat of hydration and the releasing rate of the cement paste, with or without PCMs.

The results of the heat of the hydration of the thermal energy storage cement paste are shown in Figure 6. For both OPC and TESCP, the hydration heat amounts increased with the increase in time. The CPCM can melt during the hydration process and will store thermal energy through an endothermic melting reaction. Briefly, the influence of CPCM on the cement hydration heat is believed to be a function of dilution, namely, a reduction in the volume of cement paste and supplementary effects possibly related to both sensible and latent heat absorption [30]. Hence, the total hydration heat released by the TESCP would be less. 

Figure 6 shows the heat of hydration along with the rate of heat of hydration dQ/dt of cement paste with 25% CPCM (EG-Paraffin and GNP-Paraffin) in the first 72 h. Six stages can be observed: (S0) rapid dissolution, (S1) first deceleration, (S2) induction or dormancy, (S3) acceleration, (S4) second deceleration, and (S5) slow continued reaction [34]. It can be seen that, during the first three hours, the hydration rates of CPCM cement paste are close to the control sample (without CPCM). From three hours onwards, the samples began to enter the acceleration period. Compared with that of the OCP, the heat-releasing rate in Stage S3 of the TESCP is lower and right-shifted due to the large thermal storage capacity of CPCM [30]. The high heat capacity of CPCM in cement pastes mainly results in a right-shifting phenomenon. It can also be observed that the hydration heat releasing rate during the first 72 h was lower, so thermal energy storage cement paste can be utilized in larger sections to reduce the temperature stresses, resulting from the heat of the hydration of the cement paste in the early stage and thereby decreased the early-age cracking. From this viewpoint, CPCMs are good in improving the durability properties of cement-based materials. Šavija and Schlangen [35] reported that latent heat energy storage capacity of microencapsulated PCMs helped in preventing volume changes and microcracks caused by thermal stresses in concrete. Hence, the application of TESCP into an energy pile would improve the thermal energy storage capacity and the durability of the pile structure. With the increase in the percentage of PCM contained by the carrier, the effect of PCM on the heat of hydration becomes greater. It is worth mentioning here that the rate and the amount of the heat of hydration are very important for structures having considerable mass. 

## 4. Conclusions

From an experimental investigation, the following conclusions can be drawn.

1. The compressive strength of GNP-Paraffin cement-based composites at 28 days was more than 25 MPa and hence can open an opportunity for structural purposes. Moreover, with increases in the percentage of CPCM in cement paste, the flexural strength and density of thermal energy storage cement paste composite decreased. The carbon-based CPCMs were well dispersed in the cement paste, depicting the uniform mixing of CPCM during mixing. This would help the geothermal energy in the piles to be stored evenly.
2. From infrared thermal image analysis results, the thermal-regulating performance of cement paste with carbon-based PCM was found to be superior. Moreover, in comparison to EG-Parffin-20, the GNP-Paraffin-10 showed better thermal-regulatory performance due to a higher thermal conductivity coefficient of GNP. The temperature controlling capacity of PCM in CPCM gradually reduced when the temperature exceeded the melting point of PCM.
3. The heat of the hydration results showed that carbon-based PCMs are not only more efficient in lowering the total hydration heat of cement paste but are also effective in lowering the hydration heat releasing rate. Therefore, thermal energy storage cement paste can be utilized in larger sections such as dams to reduce the undesirable tensile stresses resulting from the heat of the hydration of the cement paste.

## Figures and Tables

**Table 1:** Chemical composition of cement (expressed as oxides).

Oxides (wt %)	CaO	SiO_2_	Al_2_O_3_	Fe_2_O_3_	K_2_O	MgO	TiO_2_	Si/Ca
OPC	64.6	20.9	6.10	3.10	---	1.00	---	0.327

**Table 2:** Mix proportion (mass ratio) of composite phase change material (CPCM) in cement paste.

Cement Paste Type	Cement	Water	CPCMs (EG-PCM or GNP-PCM)	Superplasticizer (wt %)
OCP (control)	1	0.35	0	0.15
C-EG/PCM-10	1	0.35	0.1	0.3
C-EG/PCM-20	1	0.35	0.2	0.45
C-GNP/PCM-10	1	0.35	0.1	0.3
C-GNP/PCM-20	1	0.35	0.2	0.45

Note: C-XX-YY, XX means the type of CPCM; YY is percentage of CPCM.

**Table 3:** The mechanical properties of cement pastes with the CPCM.

Cement Paste Type	28-Day Compressive Strength		28-Day Flexural Strength		Density	
	Value (MPa)	% Reduction	Value (MPa)	% Reduction	Value (kg/m^3^)	% Reduction
Control OCP	66.1	/	7.9	------	2471	------
C-EG-PCM-10	14.6	77.9%	2.1	73.4%	1783.6	27.81
C-EG-PCM-20	9.0	86.4%	1.3	83.5%	1735.6	29.76
C-GNP-PCM-10	37.0	44%	5.4	31.6%	2297.6	7.02
C-GNP-PCM-20	25.6	61.3%	4.6	41.8%	2117	14.33

**Table 4:** Temperature differences between cement pastes with and without the CPCM.

Sample No.	Δ(*T*_average_)				
	0 min	3 min	6.05 min	7.02 min	14 min
C-EG/PCM-10	−0.1	−0.5	−1.8	−1.9	−1.2
C-EG/PCM-20	−0.1	−1.3	−2.6	−2.8	−1.4
C-GNP/PCM-10	0.0	−0.5	0.0	0.1	−0.3
C-GNP/PCM-20	−0.2	−1.6	−1.9	−2.0	−1.8
**Sample No.**	**Δ(*T*_spot_)**				
	**0 min**	**3 min**	**6.05 min**	**7.02 min**	**14 min**
C-EG/PCM-10	0.1	−0.8	−1.8	−2.1	−1.0
C-EG/PCM-20	1.0	−1.4	−2.5	−2.9	−1.6
C-GNP/PCM-10	−1.4	−0.7	−0.1	0.0	−0.1
C-GNP/PCM-20	−1.1	−1.6	−1.8	−2.1	−1.7

## Abbreviations

PCM	phase change material
CPCM	composite phase change material
EG	expanded graphite
GNP	graphene nanoplatelet
EG-PCM	expanded graphite-based PCM
GNP-PCM	graphene nanoplatelet-based PCM
TES	thermal energy storage
TESCP	thermal energy storage cement paste
OCP	ordinary cement paste

## References

[1] Cui H., Liao W., Memon S., Dong B., Tang W. (2014). Thermophysical and Mechanical Properties of Hardened Cement Paste with Microencapsulated Phase Change Materials for Energy Storage. Materials.

[2] Gruber M., Trüschel A., Dalenbäck J.-O. (2015). Energy efficient climate control in office buildings without giving up implementability. App. Energy.

[3] Ionescu C., Baracu T., Vlad G.-E., Necula H., Badea A. (2015). The historical evolution of the energy efficient buildings. Renew. Sustain. Energy Rev..

[4] Liu Z., Zhang L., Gong G., Li H., Tang G. (2015). Review of solar thermoelectric cooling technologies for use in zero energy buildings. Energy Build..

[5] Tyagi V., Pandey A., Buddhi D., Kothari R. (2016). Thermal performance assessment of encapsulated PCM based thermal management system to reduce peak energy demand in buildings. Energy Build..

[6] Virgone J., Giroux-Julien S. Modeling of an Active Facade Containing Phase Change Materials. Proceedings of the Conférence CLIMAMED.

[7] Agyenim F. (2016). The use of enhanced heat transfer phase change materials (PCM) to improve the coefficient of performance (COP) of solar powered LiBr/H_2_O absorption cooling systems. Renew. Energy.

[8] Zhang D., Tian S., Xiao D. (2007). Experimental study on the phase change behavior of phase change material confined in pores. Sol. Energy.

[9] Memon S.A. (2014). Phase change materials integrated in building walls: A state of the art review. Renew. Sustain. Energy Rev..

[10] Memon S.A., Lo T.Y., Barbhuiya S.A., Xu W. (2013). Development of form-stable composite phase change material by incorporation of dodecyl alcohol into ground granulated blast furnace slag. Energy Build..

[11] Zalba B., Marín J.M.A., Cabeza L.F., Mehling H. (2003). Review on thermal energy storage with phase change: Materials, heat transfer analysis and applications. Appl. Therm. Eng..

[12] Bentz D.P., Turpin R. (2007). Potential applications of phase change materials in concrete technology. Cem. Concr. Compos..

[13] Baetens R., Jelle B.P., Gustavsen A. (2010). Phase change materials for building applications: A state-of-the-art review. Energy Build..

[14] Geothermal Piles. http://www.esru.strath.ac.uk/EnvEng/Web_sites/11-12/GROUP2/MyWebSpace/piles.html.

[15] Gashti E.H.N., Uotinen V.M., Kujala K. (2014). Numerical modelling of thermal regimes in steel energy pile foundations: A case study. Energy Build..

[16] Donna A.D., Dupray F., Laloui L. (2013). Numerical study of the heating-cooling effects on the geotechnical behaviour of energy piles. Coupled Phenomena in Environmental Geotechnics.

[17] Mimouni T., Laloui L. (2015). Behaviour of a group of energy piles. Can. Geotech. J..

[18] Chen Y.-J., Nguyen D.-D., Shen M.-Y., Yip M.-C., Tai N.-H. (2013). Thermal characterizations of the graphite nanosheets reinforced paraffin phase-change composites. Compos. Part A Appl. Sci. Manuf..

[19] Zhang Z., Shi G., Wang S., Fang X., Liu X. (2013). Thermal energy storage cement mortar containing n-octadecane/expanded graphite composite phase change material. Renew. Energy.

[20] Hunger M., Entrop A.G., Mandilaras I., Brouwers H.J.H., Founti M. (2009). The behavior of self-compacting concrete containing micro-encapsulated Phase Change Materials. Cem. Concr. Compos..

[21] Xu B., Li Z. (2013). Paraffin/diatomite composite phase change material incorporated cement-based composite for thermal energy storage. Appl. Energy.

[22] Memon S.A., Lo T.Y., Shi X., Barbhuiya S., Cui H. (2013). Preparation, characterization and thermal properties of Lauryl alcohol/Kaolin as novel form-stable composite phase change material for thermal energy storage in buildings. Appl. Therm. Eng..

[23] Cui H., Tang W., Qin Q., Xing F., Liao W., Wen H. (2017). Development of structural–functional integrated energy storage concrete with innovative macro-encapsulated PCM by hollow steel ball. Appl. Energy.

[24] Farnam Y., Krafcik M., Liston L., Washington T., Erk K., Tao B., Weiss J. (2016). Evaluating the Use of Phase Change Materials in Concrete Pavement to Melt Ice and Snow. J. Mater. Civ. Eng..

[25] Zhang H., Xing F., Cui H.-Z., Chen D.-Z., Ouyang X., Xu S.-Z., Wang J.-X., Huang Y.-T., Zuo J.-D., Tang J.-N. (2016). A novel phase-change cement composite for thermal energy storage: Fabrication, thermal and mechanical properties. Appl. Energy.

[26] Silakhori M., Fauzi H., Mahmoudian M.R., Metselaar H.S.C., Mahlia T.M.I., Khanlou H.M. (2015). Preparation and thermal properties of form-stable phase change materials composed of palmitic acid/polypyrrole/graphene nanoplatelets. Energy Build..

[27] Huang X., Guo J., Gong Y., Li S., Mu S., Zhang S. (2017). In-situ preparation of a shape stable phase change material. Renew. Energy.

[28] Lin L.I., Dongxu L.I., Zhang Y., Zhang S. (2016). Preparation and characteristics of ternary fatty acid/expanded perlite composite phase change materials. J. Nanjing Tech. Univ..

[29] Xu B., Ma H., Lu Z., Li Z. (2015). Paraffin/expanded vermiculite composite phase change material as aggregate for developing lightweight thermal energy storage cement-based composites. Appl. Energy.

[30] Fernandes F., Manari S., Aguayo M., Santos K., Oey T., Wei Z., Falzone G., Neithalath N., Sant G. (2014). On the feasibility of using phase change materials (PCMs) to mitigate thermal cracking in cementitious materials. Cem. Concr. Compos..

[31] Meshgin P., Xi Y. (2012). Effect of Phase-Change Materials on Properties of Concrete. ACI Mater. J..

[32] Meshgin P., Xi Y., Li Y. (2012). Utilization of phase change materials and rubber particles to improve thermal and mechanical properties of mortar. Constr. Build. Mater..

[33] Yang J., Zhang E., Li X., Zhang Y., Qu J., Yu Z.-Z. (2016). Cellulose/graphene aerogel supported phase change composites with high thermal conductivity and good shape stability for thermal energy storage. Carbon.

[34] Cui H., Yang S., Memon S. (2015). Development of Carbon Nanotube Modified Cement Paste with Microencapsulated Phase-Change Material for Structural–Functional Integrated Application. Int. J. Mol. Sci..

[35] Šavija B., Schlangen E. (2016). Use of phase change materials (PCMs) to mitigate early age thermal cracking in concrete: Theoretical considerations. Constr. Build. Mater..

